# A novel histone deacetylase inhibitor W2A-16 improves the barrier integrity in brain vascular endothelial cells

**DOI:** 10.3389/fncel.2024.1368018

**Published:** 2024-07-19

**Authors:** Yasuteru Inoue, Yingxue Ren, Shuwen Zhang, Michael Bamkole, Naeyma N. Islam, Manikandan Selvaraj, Wenyan Lu, Thomas R. Caulfield, Yonghe Li, Takahisa Kanekiyo

**Affiliations:** ^1^Department of Neuroscience, Mayo Clinic, Jacksonville, FL, United States; ^2^Department of Quantitative Health Sciences, Mayo Clinic, Jacksonville, FL, United States; ^3^Department of Quantitative Health Sciences, Mayo Clinic, Rochester, MN, United States; ^4^Center for Regenerative Biotherapeutics, Mayo Clinic, Jacksonville, FL, United States

**Keywords:** blood–brain barrier (BBB), histone deacetylases (HDAC) inhibitor, endothelial cells (ECs), matrisome, extracellular matrix (ECM), induced pluripotent stem (IPS) cell, RNA sequencing (RNA-Seq), weighted gene co-expression network analyses (WGCNA)

## Abstract

The maturation of brain microvascular endothelial cells leads to the formation of a tightly sealed monolayer, known as the blood–brain barrier (BBB). The BBB damage is associated with the pathogenesis of age-related neurodegenerative diseases including vascular cognitive impairment and Alzheimer’s disease. Growing knowledge in the field of epigenetics can enhance the understanding of molecular profile of the BBB and has great potential for the development of novel therapeutic strategies or targets to repair a disrupted BBB. Histone deacetylases (HDACs) inhibitors are epigenetic regulators that can induce acetylation of histones and induce open chromatin conformation, promoting gene expression by enhancing the binding of DNA with transcription factors. We investigated how HDAC inhibition influences the barrier integrity using immortalized human endothelial cells (HCMEC/D3) and the human induced pluripotent stem cell (iPSC)-derived brain vascular endothelial cells. The endothelial cells were treated with or without a novel compound named W2A-16. W2A-16 not only activates Wnt/β-catenin signaling but also functions as a class I HDAC inhibitor. We demonstrated that the administration with W2A-16 sustained barrier properties of the monolayer of endothelial cells, as evidenced by increased trans-endothelial electrical resistance (TEER). The BBB-related genes and protein expression were also increased compared with non-treated controls. Analysis of transcript profiles through RNA-sequencing in hCMEC/D3 cells indicated that W2A-16 potentially enhances BBB integrity by influencing genes associated with the regulation of the extracellular microenvironment. These findings collectively propose that the HDAC inhibition by W2A-16 plays a facilitating role in the formation of the BBB. Pharmacological approaches to inhibit HDAC may be a potential therapeutic strategy to boost and/or restore BBB integrity.

## Introduction

The blood–brain barrier (BBB) is a tightly sealed monolayer with high barrier integrity formed by the brain microvascular endothelial cells covered by capillary basement membranes, pericytes and astrocyte end-feet (Abbott and Romero, 1996). Brain endothelial cells are connected by specialized tight junction proteins such as occludins and claudins, forming a high resistance paracellular barrier (Furuse, 2010; Wong et al., 2013; Hannocks et al., 2018). In addition to tight junctions, there are adherens junctions with vascular endothelial (VE)-cadherin, and pan-endothelial marker with platelet endothelial cell adhesion molecule-1 (PECAM-1) between endothelial cells (Winkler et al., 2011). These BBB junctional molecules connect adjacent endothelial cells to prevent toxins and pathogens from entering the brain, regulating the essential nutrients and metabolites (Abbott et al., 2010; Profaci et al., 2020). The BBB disfunction is characterized by reduction in junctional molecules (Morita et al., 1999; Bauer et al., 2014), leading to harmful plasma protein leakage such as fibrinogen and plasmin protease into the brain parenchyma resulting in glial activation, demyelination, and neurodegeneration (Knox et al., 2022), underscoring the detrimental effects on brain function. Indeed, the BBB damage is associated with the pathogenesis of age-related neurodegenerative diseases including vascular cognitive impairment and Alzheimer’s disease (Sweeney et al., 2018; Lin et al., 2021).

Considering the absence of therapeutics for restoration of impaired BBB integrity, targeting the mechanism of maintaining BBB integrity is an attractive strategy. However, the molecular mechanisms underpinning BBB maintenance and governing gene transcription remain elusive. We have developed a novel Wnt activator named W2A-16 (Li et al., 2022), which is documented in World Intellectual Property Organization (WIPO) PatentScope database (WO2021007313). We have reported that W2A-16 effectively mitigates amyloid pathology and cognitive decline in 5xFAD amyloid model mice. Furthermore, we have showed that W2A-16 reduces tau phosphorylation and neuroinflammation in PS19 tauopathy mice (Li et al., 2022). Moreover, our recent unpublished data indicate that W2A-16 is also a novel potent class I HDAC inhibitor. Here, we demonstrate that W2A-16 enhances BBB integrity in human brain vascular endothelial cells, primarily through its inhibitory effects on HDAC and upregulation of BBB-related molecules. Our findings show the novel compound W2A-16 may pave the way for developing promising therapeutic strategies for BBB dysfunction in various pathogenic conditions.

## Materials and methods

### Cultures of hCMEC/D3 cells

Immortalized human cerebral microvascular endothelial cells hCMEC/D3 (MilliporeSigma, Cat No. SCC066, Burlington, MA, United States) between passage 7 and 10 were used in all studies. The cells were seeded at 100,000 cells/ cm^2^ on rat collagen type I (MilliporeSigma, Cat No. 08–115) -coated plates and grown in EndoGRO^™^-MV Complete Media Kit (MilliporeSigma, Cat No. SCME004) supplemented with 1 ng/ mL FGF-2 (MilliporeSigma, Cat No. 01–106). The cells were cultured under humidified conditions at 37°C in 5% CO_2_. The culture medium was changed every other day until the cells reached 90% confluency, at which point they were subsequently used for experiments.

### W2A-16 treatment

W2A-16 was dissolved in dimethyl sulfoxide (DMSO) (BioWorld, Cat No. 40470005–2, Dublin, OH, United States) to a stock concentration of 20 mM. W2A-16 was further diluted in phosphate-buffered saline (PBS) to 500 μM prior to use in cell culture. Twenty-four hours after seeding, hCMEC/D3 cells and BMECs were treated with or without W2A-16. As control compounds, we used CI-994 (Selleckchem, Cat No. S2818, Houston, TX, United States), an HDAC inhibitor in the present study. DMSO diluted in PBS (0.025% DMSO, corresponding to the amount contained in 500 nM of W2A-16) was used for vehicle control.

### Hydrogen peroxide treatment

The hCMEC/D3 cells were cultured following the same method as described above, and hydrogen peroxide (H_2_O_2_) (Sigma-Aldrich, Cat No. H1009, St. Louis, MI, United States) was applied at a final concentration of 250 μM for a duration of 24 h. Subsequent to the H_2_O_2_ treatment, the medium was switched to cell culture medium with or without W2A-16, and additional treatment was continued for 48 h.

### Measurement of TEER in *in vitro* BBB model

**Figure 1:**
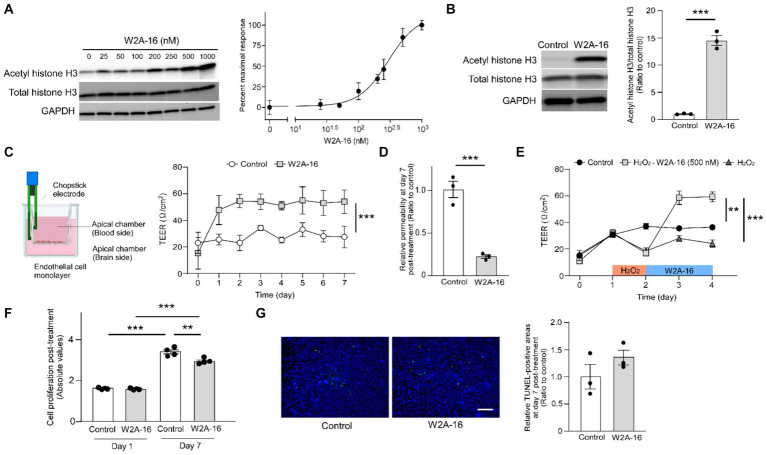
W2A-16 administration improves endothelial barrier integrity in hCMEC/D3 cells. **(A)** Dose-dependent effects of W2A-16 on histone H3 acetylation after 24 h of treatment in hCMEC/D3 cells and concentration-responsive curve of W2A-16 on histone H3 acetylation levels mediated by HDAC inhibition. The levels of acetylated histone H3 were quantified by Western blotting and normalized to those of GAPDH (*n* = 3/ group). **(B)** Histone deacetylase activity was assessed by measuring acetylated histone H3 levels through Western blotting after the administration with W2A-16 (500 nM) or vehicle control for 7 days in hCMEC/D3 cells. The levels of acetylated histone H3 were quantified by Western blotting and normalized to those of GAPDH (*n* = 3/ group). **(C)** hCMEC/D3 cells were cultured on the trans-well chamber in the presence of control or W2A-16 (500 nM) (created with BioRender.com). TEER was monitored daily for 7 days after the initiation of administration (*n* = 4/ group). Differences between groups were assessed using two-way ANOVA. **(D)** The relative permeability of FITC-dextran (70 kDa) across hCMEC/D3 cell monolayers was measured 7 days after the initiation of administration with W2A-16 (500 nM) or vehicle control. **(E)** hCMEC/D3 cells were treated with W2A-16 (500 nM) or vehicle control for 48 h, following a 24-h hydrogen peroxide (H_2_O_2_) treatment (*n* = 4/ group). TEER values on Day 4 were compared by one-way ANOVA followed by Tukey’s *post hoc* test. **(F)** Cell proliferation in hCMEC/D3 cells at 1 day and 7 days post-treatment with W2A-16 or control was assessed by colorimetric BrdU incorporation assay. **(G)** hCMEC/D3 cells were subjected to TUNEL staining, and DAPI counterstaining was performed following administration with W2A-16 (500 nM) or vehicle control for 7 days. The quantification of apoptotic cells was performed by measuring the ratio of TUNEL-positive cell areas to DAPI-positive areas. Scale bars: 100 μm. As vehicle control, 0.025% DMSO was used. Data are plotted as mean ± S.E.M. The two groups were compared by two-tailed Student’s t test unless otherwise specified. ^*^p < 0.05, ^**^p < 0.01, ^***^p < 0.001.

To evaluate the endothelial monolayer barrier integrity, the trans-endothelial electrical resistance (TEER) values were measured every day for 1 week using an EVOM3 instrument (Precision Instruments, Sarasota, FL, United States) and STX2 electrode (Precision Instruments) (Figure 1C). The hCMEC/D3 cells and BMECs were seeded in a 24-well plate with 0.4-um pore polyethylene terephthalate (PET)-inserted trans-well chamber (Greiner Bio-One, Cat No. 662641, Monroe, NC, United States). The TEER value of blank trans-well inserts was subtracted from the measured TEERs values and shown as ohm × cm^2^.

### Trans-well permeability assay

The hCMEC/D3 cells were plated on 24-well with 0.4-um pore PET-inserted trans-well chamber. After receiving W2A-16 treatment as described earlier, the cells were subjected to permeability assay. The upper chamber was filled with 300 μL of 1 mg/mL FITC-dextran (70 kDa) while the bottom plate well was replaced with 700 μL of fresh culture medium. Samples were collected from the bottom plate well at 30 min for fluorescence measurement at 485 nm excitation and 535 nm emission wavelength using a microplate reader (SpectraMax M5, Molecular Devices, United States).

### Human iPSC-derived vascular endothelial cells

Human iPSC-derived vascular endothelial cells were differentiated as previously described with modifications (Neal et al., 2019). In brief, two human iPSC lines from a normal individuals from female (iPSC line 1: mc0192) (Zhao et al., 2021) and male (iPSC line 2; iPSC) Neurodegenerative Disease initiative. Characterization of the parental KOLF2.1 J line has been published previously) (Pantazis et al., 2022) were disassociated to single cells using Accutase (Stem Cell Technologies, Cat No. 07920, Vancouver, Canada) and reseeded at 1.58 × 10^4^/ cm^2^ on Matrigel-coated plates in mTeSR1 supplemented with 10 μM Y27632 (Stem Cell Technologies, Cat No. 72302) treatment for the first 24 h. To initiate the differentiation, medium was changed to E6 medium (Thermo Fisher Scientific, Cat No. A1516501, Waltham, MA, United States) at Day 0 and daily medium changes were done. On Day 5, the medium was removed, and cells were switched to hECSR1 medium (Human Endothelial SFM (Thermo Fisher Scientific, Cat No. 11111044) supplemented with basic fibroblast growth factor (FGF) (20 ng/ ml) (PeproTech, Cat No. 100-18B, Rocky Hill, NJ, USA), 10 μM retinoic acid (Sigma-Aldrich, Cat No. R2625), and 1X B27 (Thermo Fisher Scientific, Cat No. 17504044). Cells were reseeded using a ratio of 1 well of a 6-well plate to 6 inserts of 24 trans-well, or 6 wells of a 24-well plate. Cells were maintained in hECSR2 24 h after subculture (hECSR1 lacking retinoic acid and basic fibroblast growth factor).

### Immunostaining of vascular cells

For immunostaining, cells were fixed in 4% paraformaldehyde for 15 min then washed with PBS three times. After fixation, cells were permeabilized with PBS containing 0.3% Triton X-100 (Sigma-Aldrich, Cat No. X100) and blocked with Protein Block Serum-Free Ready-To-Use (Agilent Technologies, Cat No. X0909, Santa Clara, CA, United States). Cells were incubated with primary antibodies in background-reducing dilution buffer (Agilent Technologies, Cat No. S3022) overnight at 4°C with the following primary antibodies: mouse monoclonal anti-ZO-1 (1: 200 dilution; Invitrogen, Cat No. 33–9,100, Carlsbad, CA, United States, clone ZO1-1A12), mouse monoclonal anti-claudin-5 (1:200 dilution; Invitrogen, Cat No. 35–2,500, clone 4C3C2), mouse monoclonal anti-CD31 (1:50 dilution; R&D Systems, Cat No. BBA7, Minneapolis, USA, clone 9G11), rabbit polyclonal anti-occludin (1:400 dilution; Proteintech Group, Cat No. 27260-1-AP, Chicago, IL), mouse monoclonal anti-VE-cadherin (1:50 dilution; Cat No. sc-9989, Santa Cruz Biotechnology, Santa Cruz, CA, United States), mouse monoclonal anti-β-catenin (1:500 dilution; BD Biosciences, Cat No. 610154, Franklin Lakes, NJ, USA), rabbit polyclonal anti-TMEM100 (1:300 dilution; Proteintech Group, Cat No. 25581-1-AP), rabbit polyclonal anti-DHH (1:300 dilution; Proteintech Group, Cat No. 13889-1-AP), rabbit polyclonal anti-PADI2 (1:300 dilution; Proteintech Group, Cat No. 12110-1-AP), mouse polyclonal anti-Annexin A1 (1:100 dilution; Novus Biologicals, Cat. No. NBP2-23485, Littleton, CO, United States), and mouse polyclonal anti-TSG6 (1:300 dilution; R&D Systems, Cat. AF2104), subsequently subjected to incubation with Alexa 488- and Alexa 568-conjugated secondary antibodies (Invitrogen) for 2 h at room temperature. The cells were counterstained with DAPI (Thermo Fisher Scientific, Cat No. 62248) at 1: 5000 in PBS for nuclei visualization. The images were captured using a Keyence BZ-X800 fluorescence microscope (Keyence, Osaka, Japan). We randomly captured images from each treatment group and used ImageJ 1.54 software (NIH, Bethesda, MD) to measure the percentage of immunopositive areas in comparison to the total areas. The images were converted to 8-bit grayscale, and the threshold function was applied to quantify the positively stained area and intensity.

### RNA isolation and real-time PCR analysis

Total RNA was isolated by using TRIzol (Invitrogen, Cat No. 15596026) followed by RNeasy Mini kit (QIAGEN, Cat No. 74104, Valencia, CA, United States) and subjected to DNase I (QIAGEN, Cat No. 79254) digestion to remove contaminating genomic DNA. For real-time PCR analysis, complementary DNA was synthesized from total RNA using iScript^™^ cDNA Synthesis Kit (Bio-Rad, Cat No. 1708890, Hercules, CA, United States). Complementary DNA was added to a reaction mix containing gene-specific primers and SsoAdvanced^™^ Universal SYBR^®^ Green Supermix (Bio-Rad, Cat No. 1725271). Real-time quantification was performed on the QuantStudio 7 Flex Real-Time PCR System (Applied Biosystems, Foster City, CA). Data were analyzed by the ΔΔCT method using QuantStudio 7 Flex Real-Time PCR System software. Predesigned primers from Integrated DNA Technologies (IDT, Coralville, CA, United States) were used to quantify the mRNA levels of *CDH5* (Hs.PT.58.4732035), *CLDN5* (Hs.PT.58.1483777.g), *OCLN* (Hs.PT.58.15235048), *PECAM1* (Hs.PT.58.19487865), and *TJP1* (Hs.PT.58.2456962). The relative gene expression was normalized to *GAPDH* (Hs.PT.39a.22214836).

### Western blotting

Cells were lysed with RIPA buffer (MilliporeSigma, Cat No. 20–188) supplemented with PhosSTOP phosphatase inhibitors (Roche, Cat No. 4906845001, Indianapolis, IN, USA) and complete protease inhibitors (Roche, Cat No. 11697498001). After centrifugation (15,000 rpm for 15 min at 4°C), the supernatant was used for western blotting. Total protein concentrations were determined using a Pierce^™^ BCA Protein Assay Kits (Thermo Fisher Scientific, Cat No. 23225). An equal amount of protein for each sample was loaded onto SDS-PAGE gel and transferred onto PVDF membranes (MilliporeSigma, Cat No. IPVH00010). After blocking, we immunostained the transferred proteins overnight at 4°C with the following primary antibodies: mouse monoclonal anti-ZO-1 (1:1,000 dilution; Cat No. 33–9,100, Invitrogen, clone ZO1-1A12), mouse monoclonal anti-occludin antibody (1:200 dilution; Invitrogen, Cat No. 33–1,500, clone OC-3F10), mouse monoclonal anti-CD31 (1:500 dilution; R&D Systems, Cat No. BBA7), rabbit polyclonal anti-claudin 5 (1:1000 dilution; Milliporesigma, Cat No. ABT45), mouse monoclonal anti-VE-cadherin (1:500 dilution; Santa Cruz Biotechnology, Cat No. sc-9989), rabbit anti-monoclonal Acetyl-Histone H3 (Lys9) (1: 1,000 dilution; Cell Signaling, Cat No. 9649S, Danvers, MA, United States), rabbit monoclonal anti-Histone H3 (1: 2,000 dilution; Cell Signaling, Cat No. 4499S), mouse anti-monoclonal β-catenin (1:1,000 dilution; BD Biosciences, Cat No. 610154), rabbit monoclonal anti-non-phospho (active) β-catenin (Ser45) (1:500; Cell Signaling, Cat No. 19807S), and anti-GAPDH antibody (1: 2,000 dilution; Cell Signaling, Cat No. 2118S). The membrane was then probed with HRP-conjugated secondary antibodies (Abcam, Cat No. ab6721 and ab6789, Cambridge, MA, United States) and visualized using the ChemiDoc MP Imaging System (Bio-Rad). We used ImageJ 1.54 software (NIH, Bethesda, MD) to analyze the intensity of specific bands.

### BrdU incorporation assay

Cellular proliferation was assessed by BrdU incorporation assay according to the manufacturer’s instructions (Abcam, Cat No. ab126556). Briefly, hCMEC/D3 cells were plated at 33,000 cells/ well, 100 μL/ well in 96-well plate overnight. Following a 24-h incubation period, W2A-16 were administrated and further incubated for 24 and 168 h. Throughout the treatment period, the culture medium was refreshed daily. After treatment, BrdU reagent was diluted in culture medium in each well and incubated with cells for 18 h. After incubation with anti-BrdU detector antibody followed by peroxidase goat anti-mouse IgG, tetramethylbenzidine-peroxidase substrate was added. The substrate-peroxidase reaction was stopped with stop solution, and the signal was read at a wavelength of 450 nm.

### TUNEL staining

Apoptosis was assessed by the terminal deoxynucleotidyl transferase-mediated dUTP nick end labelling (TUNEL) staining (Roche, Cat No. 11684795910) according to the manufacturer’s protocol. Following DAPI staining, images were captured using a Keyence BZ-X800 fluorescence microscope.

### HDAC activity assay

The hCMEC/D3 cells were lysed in RIPA lysis buffer (MilliporeSigma). Lysates were subsequently used for HDAC activity assay using fluorometric HDAC activity assay kit (Abcam, Cat No. ab156064) according to the manufacturer’s protocol. We conducted fluorescence measurements using a microplate reader (SpectraMax M5, Molecular Devices) at an excitation wavelength of 355 nm and an emission wavelength of 460 nm.

### RNA -sequencing and transcriptomics data analysis

Total RNA of the cells was isolated by using TRIzol RNA isolation reagents (Invitrogen), followed by DNase and Cleanup using RNase-Free DNase Set (QIAGEN) and RNeasy Mini Kit (QIAGEN). The quantity and quality of all RNA samples were measured with 2,100 Bioanalyzer (Agilent Technologies) using the Agilent RNA 6,000 Nano Chip (Agilent Technologies, Cat No. G2938-80023). A total of 15 samples (5 samples per treatment group) with RNA integrity number ≥ 9.0 were used for RNA-sequencing (RNA-seq) at Mayo Clinic sequencing core using an Illumina HiSeq 4,000. Reads were mapped to the human genome hg38. Mayo Clinic RNA-sequencing analytic pipeline, MAP-RSeq Version 3.13, was used to generate raw gene read counts, along with sequencing quality control (Kalari et al., 2014). We conducted conditional quantile normalization (CQN) on the raw gene counts to correct for GC bias, gene length differences, and global technical variations (Hansen et al., 2012). According to the bimodal distribution of the CQN-normalized and log2-transformed reads per kb per million (RPKM) gene expression values, genes with average of log2 RPKM ≥ 0 in at least one group were included in the analysis. Differential gene expression analyses were performed by Partek Genomics Suite (Partek Inc., St. Louis, MO) using CQN-normalized log2RPKM values. The Benjamini-Hochberg step-up procedure was applied to adjust for multiple testing. Differentially expressed genes (DEG) were defined by thresholds of adjusted *p* value <0.05 and |fold change| (FC) ≥ 2. Pathway analyses of differentially expressed genes were performed using Metascape^1^ (Zhou et al., 2019). To identify gene groups that are correlated with W2A-16 treatment, we performed weighted gene co-expression network analysis (WGCNA). We used the power of 12, the minimum modules size as 60, and mergeCutHeight of 0.3 to build scale-free topology using signed hybrid network. To assess the correlation of modules to treatment, we defined the control group as 0 and the W2A-16-treated group as 1. Gene ontologies of module genes were performed using R package anRichment. Gene–gene connections among top hub genes were visualized using VisANT version 5.51 (Hu et al., 2013).

### Statistical analysis

All quantified data represent an average of samples. Statistical significance was determined by two-tailed Student’s t-test and one-way ANOVA followed by Tukey’s *post hoc* test. Differences between groups (time x drug) were assessed using two-way ANOVA followed by Tukey *post hoc* corrections (GraphPad Prism software). *p* < 0.05 was considered significant. Unless otherwise specified, data were presented as mean ± standard error of the mean (SEM).

## Results

### W2A-16 enhances the barrier function of hCMEC/D3 cells via HDAC inhibition

HDAC inhibition results in histone acetylation. We determined the effects of W2A-16 on histone H3 acetylation. We found that W2A-16 enhanced H3 acetylation with an EC_50_ value of around 295 nM in hCMEC/D3 cells (Figure 1A) and set the treatment concentration of W2A-16 at 500 nM, approximately 2-fold its EC_50_ value. HDAC activity was significantly reduced following W2A-16 treatment at 500 nM (Supplementary Figure S1A). Immunoblotting analysis revealed that histone H3 was significantly acetylated following W2A-16 treatment for 7 days, although there were no differences in non-phospho β-catenin levels between W2A-16 and non-treated control group (Figure 1B; Supplementary Figure S1B). The nuclear translocation of β-catenin was not evident after W2A-16 treatment (Supplementary Figure S1C). We sought to determine whether W2A-16 could strengthen the endothelial monolayer barrier integrity of hCMEC/D3 cells and observed a significant elevation in the TEER values following the initiation of W2A-16 treatment compared with non-treated control group. Furthermore, the W2A-16 treatment group consistently exhibited elevated TEER values throughout the entire treatment period (Figure 1C), which exhibited a concentration-dependent manner, with higher TEER values of hCMEC/D3 cells treated with W2A-16 at 500 nM than those of the cells treated with W2A-16 at 250 nM (Supplementary Figure S1D). We then assessed the permeability of the hCMEC/D3 monolayer by examining the passage of FITC-dextran (70 kDa), and found that the permeability to 70 kDa dextran was reduced in the W2A-16 treatment group compared with non-treated control group (Figure 1D). We also elucidated the restorative capabilities of W2A-16 under stressed conditions. Following a 24-h treatment with hydrogen peroxide (H_2_O_2_), we observed a decrease in TEER values. Subsequent treatment with W2A-16 resulted in a dramatic increase of TEER values compared with non-treated control group (Figure 1E). We further analyzed the cell proliferation rates at Day 1 and 7 post-treatments by BrdU incorporation assay. No differences in cell viability and proliferation rates were detected between control group and W2A-16 treatment group at Day 1 post-treatment. At Day 7 post-treatment, cell proliferation rate in W2A-16 treatment group was lower than that in the non-treated control group (Figure 1F). The number of apoptotic cells, assessed through TUNEL staining at Day 7 post-treatments, showed no significant differences between control group and W2A-16 treatment group (Figure 1G).

### W2A-16 increases the expression of BBB-related molecules in hCMEC/D3 cells

**Figure 2:**
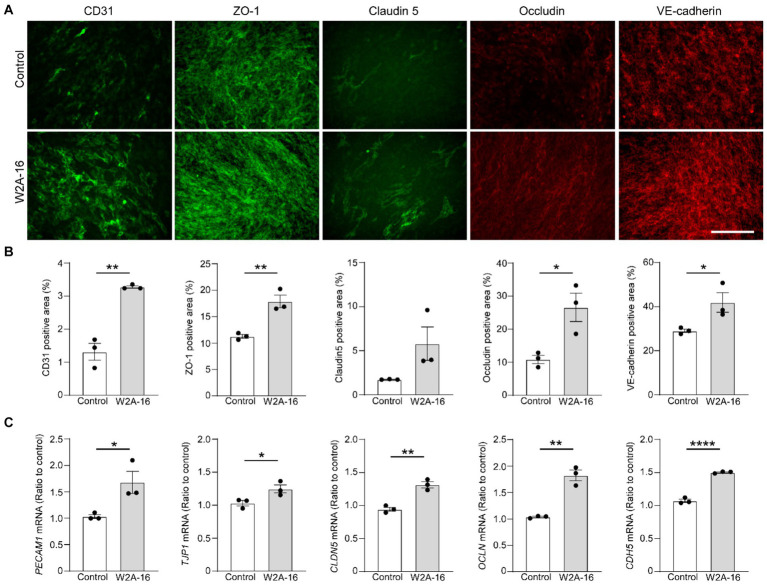
W2A-16 administration facilitates tight junction and adherens junction formation in hCMEC/D3 cells. **(A,B)** hCMEC/D3 cells were immunostained for CD31, ZO-1, Claudin-5, Occludin, and VE-Cadherin after the administration with W2A-16 (500 nM) or vehicle control for 7 days. Scale bars: 100 μm **(A)**. Percentage of immune-positive areas per 20X field for each staining were quantified by ImageJ **(B)**. **(C)** The mRNA levels of *PECAM1*, *TJP1*, *CLDN5*, *OCLN*, and *CDH5* in hCMEC/D3 cells at 7 days post-treatment with W2A-16 or control was assessed were measured by RT-qPCR. Each measurement was normalized to *GAPDH* mRNA levels. As vehicle control, 0.025% DMSO was used. Data are plotted as mean ± S.E.M. (*n* = 3/group). ^*^p < 0.05, ^**^p < 0.01, ^****^p < 0.0001 by two-tailed student *t* test.

**Figure 3:**
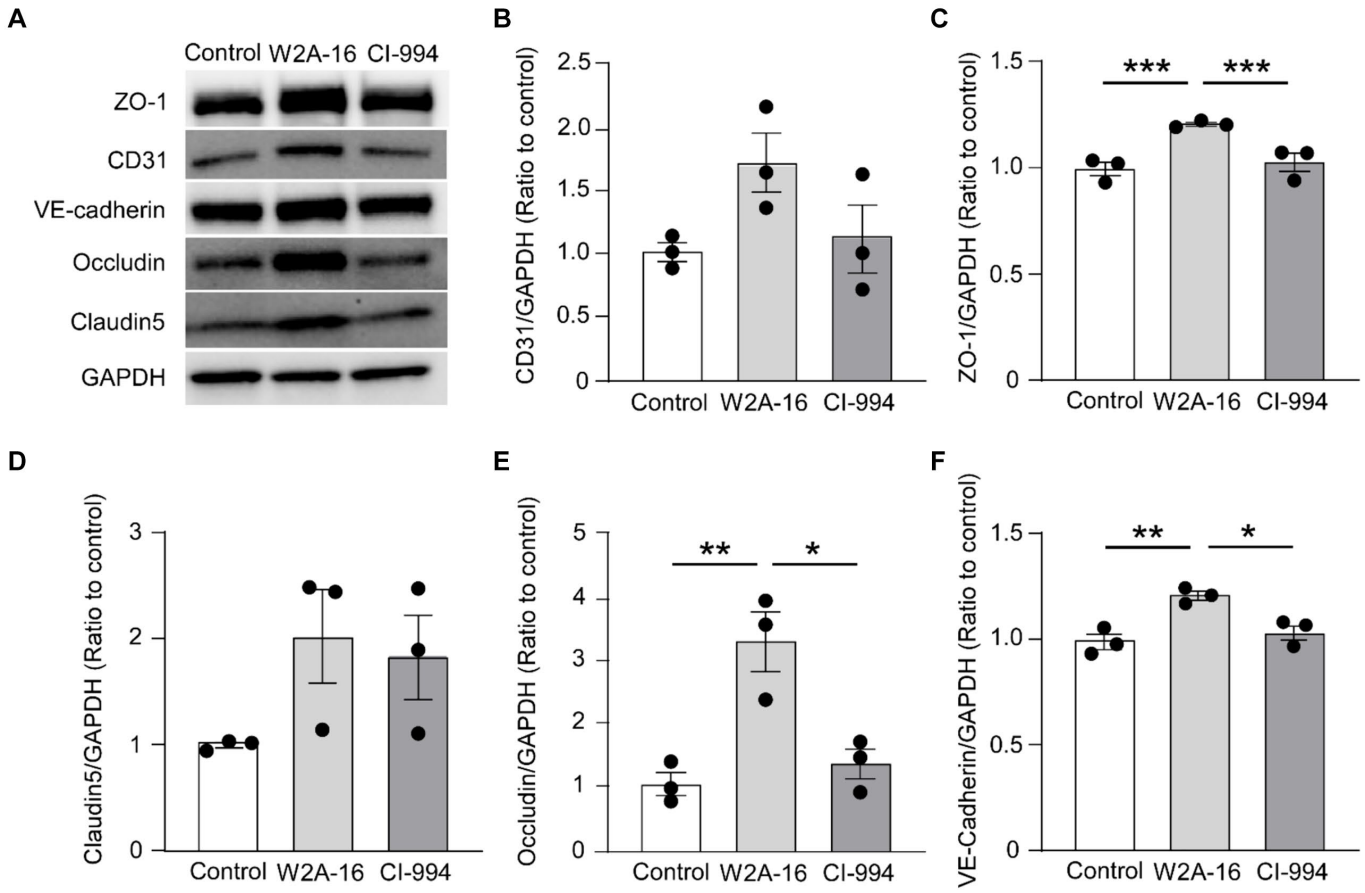
W2A-16 administration increases tight junction and adherens junction proteins in hCMEC/D3 cells. **(A)** hCMEC/D3 cells were treated with W2A-16 (500 nM), CI-994 (1 μM), or vehicle control for 7 days followed by analyses through Western blotting for BBB related molecules. **(B–F)** The protein levels of CD31 **(B)**, ZO-1 **(C)**, claudin5 **(D)**, occludin **(E)**, and VE-cadherin **(F)** were quantified Western blotting and normalized to that of GAPDH. As vehicle control, 0.025% DMSO was used. Data are plotted as mean ± S.E.M. (*n* = 3/group). ^*^p < 0.05, ^**^p < 0.01, ^***^p < 0.001 by one-way ANOVA followed by Tukey’s *post hoc* test.

To determine whether the expression of BBB-related molecules is affected by W2A-16, we performed immunocytochemical analyses for the tight junction proteins using ZO-1, Claudin-5, Occludin, adherens junction proteins of VE-Cadherin, and pan-endothelial marker of CD31. We quantified the percentage of the immune-positive areas to demonstrate that CD31-, ZO-1, Occludin-, and VE-cadherin- positive areas were significantly larger in the W2A-16 treatment group compared with non-treated control group. No significant differences were observed in the DAPI-positive areas between the W2A-16 treatment group and the non-treated control group (Figures 2A,B; Supplementary Figure S2A). Subsequently, we confirmed the increased mRNA levels of tight junction markers (*TJP1*, *CLDN5*, and *OCLN*), adherens junction markers (*CDH5*), and pan-endothelial marker (*PECAM1*) of hCMEC/D3 cells in W2A-16 treatment group compared with non-treated control group using RT-qPCR (Figure 2C). We then tested the BBB protein expression levels and found that ZO-1, Occludin, and VE-cadherin levels were increased in W2A-16 treatment group, consistent with the RT-qPCR results (Figure 3). To further characterize the effects of W2A-16, we administered CI-994, a class I HDAC inhibitor, and found that ZO-1, Occludin, and VE-cadherin levels were increased compared to the CI-994 treatment group (Figure 3), suggesting that W2A-16 is more potent in inducing the expression of BBB-related molecules.

### W2A-16 promotes extracellular matrix-associated gene expression in hCMEC/D3 cells

**Figure 4:**
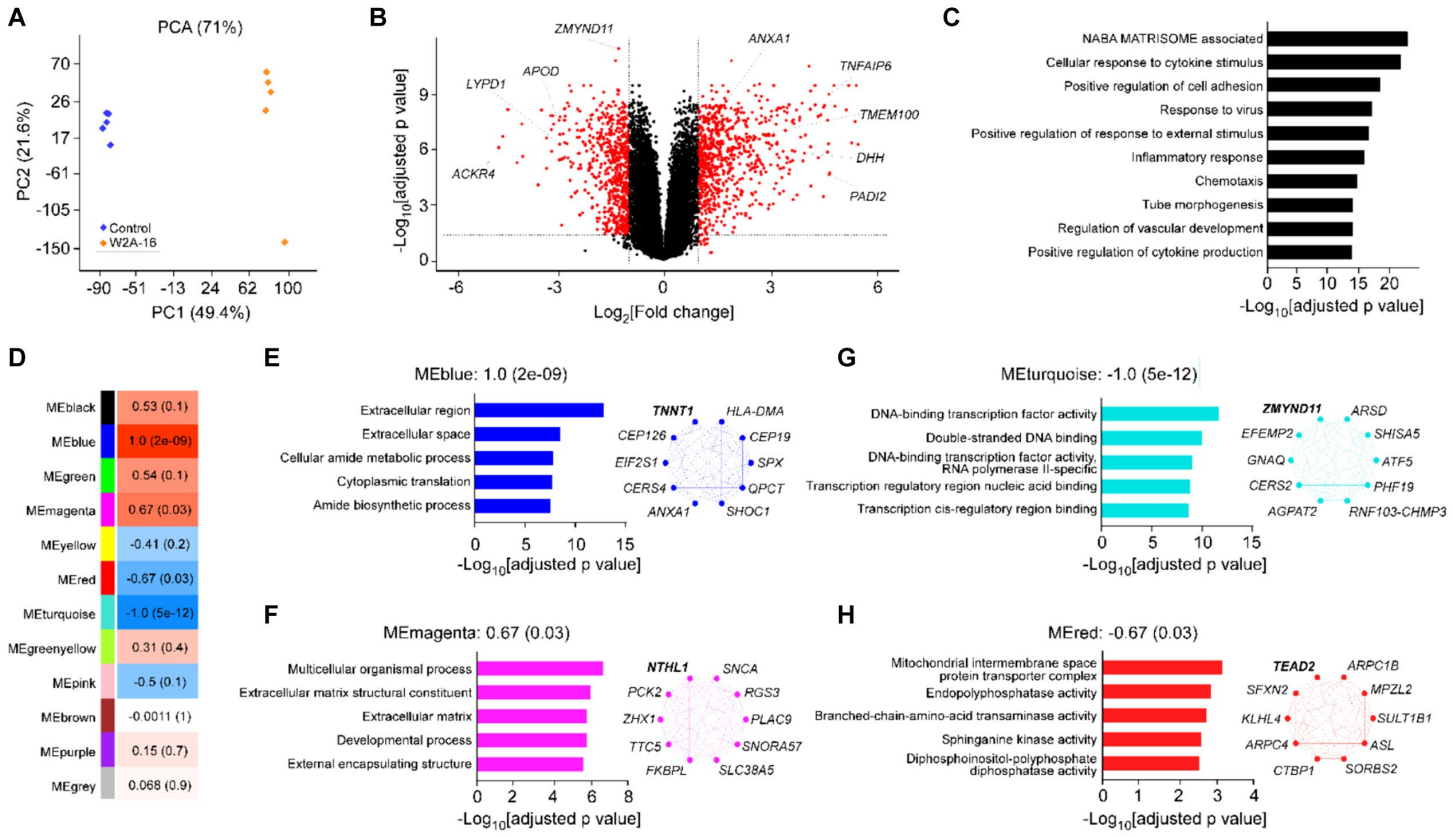
Effect of W2A-16 administration on transcriptomic profiles in hCMEC/D3 cells Bulk RNA-sequencing was performed in hCMEC/D3 cells after the administration with W2A-16 (500 nM) or vehicle control for 7 days (*n* = 5/group). **(A)** PCA of W2A-16-treated and control samples. **(B)** Volcano plot of DEGs affected by W2A-16 are shown. **(C)** Top 10 pathways through gene ontology analyses in the DEGs affected by W2A-16 (adjusted *p* value <0.05, |fold change| > 2) are shown. **(D)** Module-trait relationships between module eigengenes (MEs) and W2A-16 treatment status are shown. The correlation coefficient (r) and the correlation *p*-value in the parentheses are indicated in each module. **(E–H)** Top 5 gene ontologies and top 10 genes with the highest intramodular connectivity with W2A-16 treatment in MEblue **(E)**, MEmagenta **(F)**, MEturquoise **(G)**, and MEred **(H)** are shown. The top hub gene in each module is specified in Bold.

To obtain a detailed picture of molecular changes associated with HDAC inhibition, we analyzed endothelial cell transcripts by bulk RNA-seq. Principal component analysis (PCA) demonstrated clear separation between two treatment modalities (Figure 4A). We found 1,336 DEGs (722 upregulated and 614 downregulated) in W2A-16 treatment group compared with non-treated control group (Figure 4B). We identified “NABA matrisome associated” and “cellular response to cytokine stimulus” (−Log_10_[adjusted *p* value] > 20) as the top-ranked pathway enriched by DEG (Figure 4C). Since matrisome, consisting of extracellular matrix (ECM) proteins and associated factors, plays a crucial role in the formation and maintenance of BBB (Joutel et al., 2016), genes involved in the regulation of extracellular microenvironment and cytokines could be modulated by HDAC inhibition. Finally, we conducted weighted gene correlation network analysis (WGCNA) and identified two gene modules that were up-regulated (blue and magenta), and two gene modules that were down-regulated (turquoise and red) in the W2A-16 treatment group compared with vehicle control group (Figure 4D). Enrichment analyses revealed genes related to cellular processes encompassing “extracellular region” in blue module genes, and “structural development of extracellular matrix” in magenta module genes, indicating the involvement of W2A-16 treatment in extracellular structures and functions. In addition, “DNA binding-transcription related pathways” were in turquoise module, and genes related to “membrane enzyme activities and transport functions” were in red module (Figures 4E–H). We opted to further validate the upregulated proteins in DEGs: Annexin A1, TMEM100, PADI2, DHH, and TSG6 through immunocytochemistry. The immunopositive areas for these molecules were larger in the W2A-16 treatment group compared with non-treated control group (Supplementary Figure S2B).

### The effects of W2A-16 effect on barrier function in human iPSC-derived vascular endothelial cells

**Figure 5:**
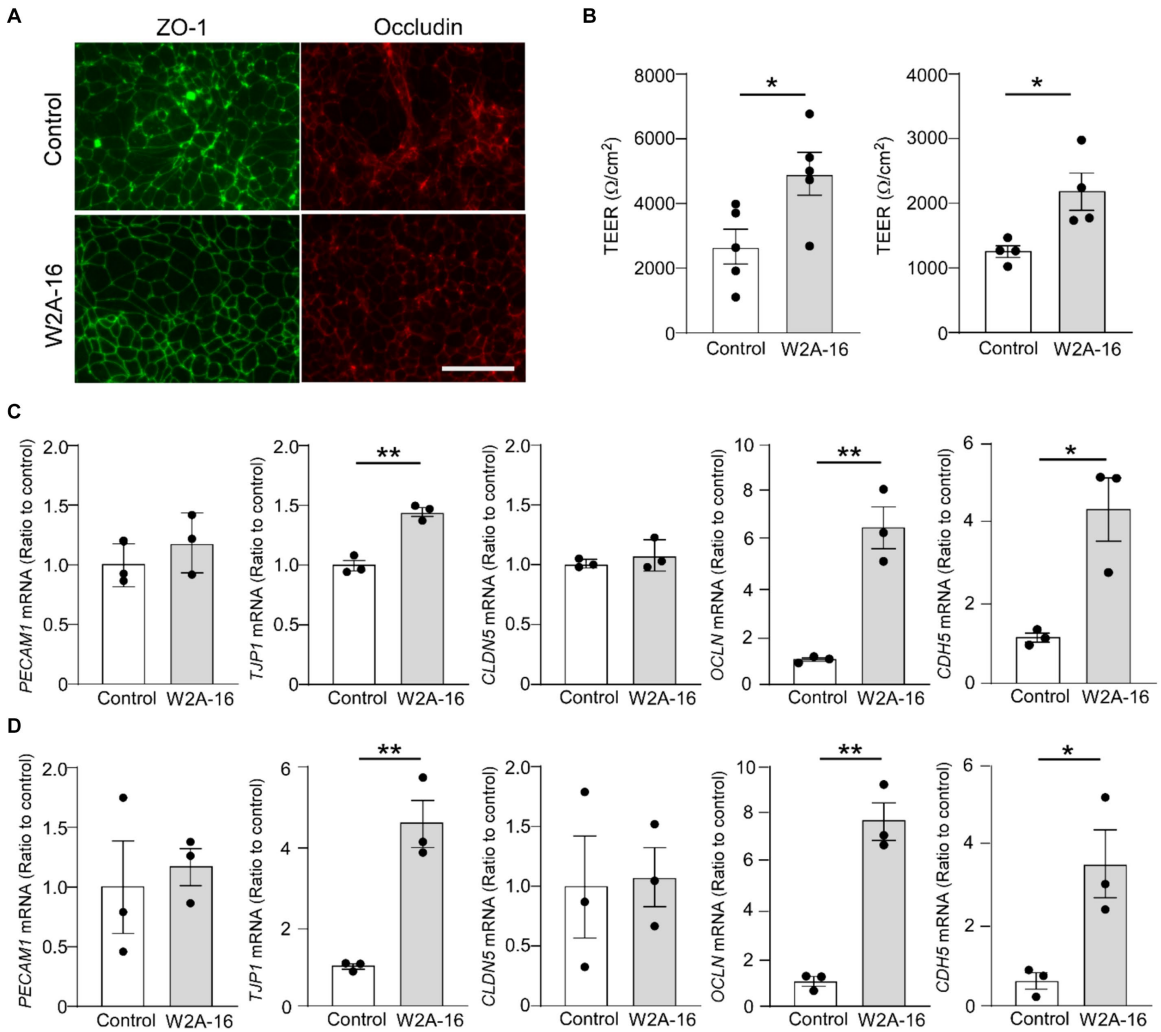
W2A-16 administration improves barrier formations in human iPSC-derived vascular endothelial cells **(A)** Representative images of immunostaining for ZO-1 and occludin in the iPSC-derived vascular endothelial cells (iPSC line 1) treated with or without W2A-16 are shown. Scale bars: 50 μm. **(B)** Human iPSC-derived vascular endothelial cells were treated with W2A-16 (500 nM) or vehicle control for 7 days and subjected to the TEER measurement. Left: iPSC line 1, Right: iPSC line 2. **(C,D)** The mRNA levels of *TJP1*, *OCLN*, and *CDH5* in iPSC-derived vascular endothelial cells at 7 days post-treatment with W2A-16 or control were measured by RT-qPCR. C: iPSC line 1, D: iPSC line 2. Each measurement was normalized to *GAPDH* mRNA levels. As vehicle control, 0.025% DMSO was used. Data are plotted as mean ± S.E.M. (*n* = 3/group). ^*^p < 0.05, ^**^p < 0.01, by two-tailed student *t* test.

We further examined whether W2A-16 treatment elicit enhancement of BBB integrity of human iPSC-derived vascular endothelial cells. The tight junction protein ZO-1 and Occludin in the treatment groups showed better organized honeycomb structures by immunohistochemical analyses (Figure 5A). We observed W2A-16-treated human iPSC-derived vascular endothelial cells showed significantly higher TEER values compared with vehicle control (Figure 5B). We next examined the mRNA expression levels and found that W2A-16 treatment upregulated the mRNA levels of *TJP1*, *OCLN*, and *CDH5* (Figures 5C,D).

## Discussion

Epigenetic modification is essential in the regulation of gene expression (Pons et al., 2009), and histone modification has been well established as key mechanism in this process (Brownell et al., 1996). Histone acetyltransferases (HATs) and HDACs are critical regulators of gene expression through histone acetylation and deacetylation, leading to relaxation and condensing of nucleosomes (Verdin and Ott, 2015). HDAC inhibitors represent potent compounds capable of inducing the histone acetylation. This process effectively neutralizes the positive charge on the histones, diminishing their affinity to the DNA molecules, leading to chromatin structure relaxation and promotes gene expression by enhancing the binding of DNA with transcription factors (Milazzo et al., 2020). Indeed, HDAC inhibitors are used for cancer treatment, inducing cell apoptosis, cell cycle arrest, and modulation of immune responses (Bolden et al., 2006). Recently, the pleiotropic nature of HDAC inhibitors has garnered attention beyond their clinical usage as cancer treatment (Hull et al., 2016). In the present study, we demonstrated that the novel compound, W2A-16 enhances and sustains barrier properties of the monolayer of endothelial cells, as evidenced by increased TEER and upregulation of the expression of BBB-related molecules compared with non-treatment controls, offering opportunities for exploration of its underpinning mechanism. Despite the dual functionality of W2A-16 as HDAC inhibitor and Wnt/β-catenin signaling activator, our results demonstrated only elevated levels of histone H3 acetylation, indicating effective HDAC inhibition. In contrast, there are no significant differences in the non-phospho β-catenin/ total β-catenin ratios between W2A-16 and non-treatment control group, and the nuclear translocation of β-catenin was not observed after W2A-16 treatment, suggesting the non-activation of the Wnt/β-catenin pathway. These findings indicate that W2A-16 at 500 nM used in the current study could induce a substantial enhancement of BBB integrity, given that the EC_50_ value of W2A-16 for histone H3 acetylation was 295 nM. We also observed a concentration-dependent elevation and restorative ability against stress-induced conditions in TEER values. However, the activation of Wnt/β-catenin signaling was not evident, suggesting that higher concentrations may be required to induce Wnt/β-catenin activation in hCMEC/D3 cells, although additional studies are warranted. Indeed, *in vitro* experiment using cultured primary human brain microvascular endothelial cells reported that increased HDAC3 activity was associate with the downregulation of junctional proteins after oxygen glucose deprivation and reoxygenation. In addition, a selective HDAC3 inhibitor RGFP966 treatment reduced the paracellular permeability and increased Claudin-5 expression (Zhao et al., 2019). Another study using rodent model of cerebral ischemia demonstrated that class IIA HDAC inhibitor TMP269 treatment protected BBB by increasing expression of the tight-junction proteins in endothelial cells (Su et al., 2020). These observations indicate that HDAC inhibition can be protective against BBB dysfunction, and our results added the evidence that HDAC inhibition contribute to enhance BBB integrity in intact endothelial cells. The ability of W2A-16 to increase BBB-related molecules is higher compared to CI-994, a known class I HDAC inhibitor, suggesting that W2A-16 is a more potent class I HDAC inhibitor. To further elucidated the mechanisms underlying the functionality of W2A-16, we conducted RNA-seq analysis to identify differentially expressed genes and enriched pathways (Ashburner et al., 2000), The top DEG-enriched pathways include “NABA matrisome associated” and “cellular response to cytokine stimulus.” The matrisome is a complex and heterogeneous network of ECM (Naba et al., 2012), classifying into “core matrisome” and “matrisome-associated” proteins. The core matrisome consists of structural ECM proteins, including proteoglycans, collagens, and glycoproteins. On the other hand, matrisome-associated proteins are subdivided into secreted proteins (cytokines and growth factors), ECM regulators (enzyme), and ECM-affiliated proteins (Hynes and Naba, 2012; Naba et al., 2012). To gain insight into gene functionalities within each pathway, we focused on *ANXA1* and *DHH* included in “NABA matrisome associated,” along with *PADI2* and *ACKR4* included in “cellular response to cytokine stimulus.” ANXA1 is expressed particular in the endothelium of the brain microvasculature (Solito et al., 2008), and recombinant ANXA1 restores cell polarity, cytoskeleton integrity, and paracellular permeability through inhibition of the small G protein RhoA (Cristante et al., 2013). Endothelial DHH expression is necessary to maintain adherens and tight junction mRNA and protein expression levels (Mora et al., 2020). Padi2 is reported to be involved in endothelial cell migration and proliferation (Khajavi et al., 2017). ACKR4 promotes endothelial-to-mesenchymal transition (EndMT) in endothelial cells, which has been reported to be associated with BBB dysfunction in neurological disorders. Therefore, substantial downregulation following W2A-16 treatment may be beneficial for BBB integrity. We next investigated the genes not included in the top-ranked pathway, manifested substantial up- or downregulation following W2A-16 treatment. TNFAIP6 is a secreted protein that binds hyaluronic acid and involved in ECM matrix stability (Mukhopadhyay et al., 2001; Fulop et al., 2003). ZMYND11 exerts its inhibitory effects on the viability, migration of endothelial cells when overexpressed (Zhu et al., 2022). *TMEM100* and *APOD* demonstrate their contradictory effect on angiogenesis, in which TMEM100 is identified to promote angiogenesis (Moon et al., 2020), whereas, APOD suppresses angiogenesis via PI3K-Akt-eNOS signaling (Lai et al., 2017). Collectively, W2A-16 appears to exert positive effects on BBB integrity by stabilizing the ECM microenvironment. In this context, matrisome-associated proteins may contribute to ECM stabilization. Notably, the present RNA-seq finding of “cellular response to cytokine stimulus” as the enriched pathway underscores the significance of cytokines and key regulators in physiological angiogenesis, as highlighted by Potente et al. (2011). The collective influence of these factors induced by W2A-16 plays a pivotal role in promoting angiogenesis and ECM maturation, leading to the BBB integrity enhancement. To investigate gene co-expression pattern changes induced by W2A-16, we also performed WGCNA to identify gene modules that were correlated with W2A-16 treatment (Zhang and Horvath, 2005). We found a top gene ontology to be which suppression of DNA binding transcriptional activity. This effect may potentially be attributed to the negative feedback resulting from enhanced transcriptional activity induced by W2A-16, and further study are warranted to elucidate the mechanism.

In the additional experiment using human BMECs, we observed the upregulation of mRNA levels of *TJP1*, *OCLN*, and *CDH5* in the BMECs. The generation of endothelial cells from human iPSCs has aroused controversies recently (Lu et al., 2021a,b). The BMECs exhibit characteristics more closely resembling epithelial cells, as indicated by their low expression of *CDH5*, which is a key marker of endothelial function. However, treatment with W2A-16 has been shown to increase the expression of *CDH5* in iPSC-derived endothelial cells, suggesting that HDAC inhibition may also have the additional potential to induce a shift towards a more endothelial-like phenotype. Conventional pan-HDAC inhibitors exhibit potential adverse effects due to the limited isoform selectivity. In terms of off-target effects, W2A-16 could harbor a favorable profile with limited off-target effects with its specificity towards class I HDACs inhibition. Several limitations of our study should be mentioned. One is that the absence of *in vivo* study, which will assess the long-term effects of W2A-16. HDAC inhibitors are recognized for their capacity to induce cell cycle arrest and apoptosis, particularly in cancer cells (Marks and Xu, 2009). While these effects are desired in cancer cell lines, their manifestation in normal cell lines may present a potential drawback. To mitigate potential cellular damage, adjustments to the duration or frequency of treatment are thus warranted. The second limitation pertains to the exclusive reliance on endothelial cell lines in the experimental setup for recapitulating BBB functionality. While the current approach serves to emulate the BBB, it is recognized that incorporating mural cells and astrocytes could render it a more representative system for studying BBB dynamics. In the present experimental setting, although the Wnt/β-catenin pathway was not explicitly activated, the potential subtherapeutic effects for BBB remain to be elucidated. Future research is warranted to investigate the molecular properties associated with Wnt/β-catenin signaling. In summary, our study suggests a novel compound W2A-16 enhances BBB integrity through upregulation of BBB-related molecules by HDAC inhibition. In addition, RNA-seq transcript profiles revealed that W2A-16 may contribute to synthesis and development of ECM. Pharmacological approaches to inhibit HDAC activity may be novel therapeutics to boost and/or restore BBB integrity.

## Data availability statement

The original contributions presented in the study are included in the article/Supplementary material.

## Ethics statement

Ethical approval was not required for the studies on humans in accordance with the local legislation and institutional requirements because only commercially available established cell lines were used.

## Author contributions

YI: Data curation, Formal analysis, Investigation, Writing – original draft. YR: Data curation, Formal analysis, Writing – original draft. SZ: Data curation, Formal analysis, Writing – original draft. MB: Data curation, Writing – original draft. NI: Resources, Writing – original draft. MS: Resources, Writing – original draft. WL: Resources, Writing – original draft. TC: Writing – review & editing, Resources. YL: Funding acquisition, Investigation, Methodology, Resources, Writing – original draft. TK: Conceptualization, Funding acquisition, Methodology, Supervision, Validation, Visualization, Writing – review & editing.

## Footnotes

^1^https://metascape.org
