# Supplementary material for: A novel histone deacetylase inhibitor W2A-16 improves the barrier integrity in brain vascular endothelial cells
Source: Front Cell Neurosci. 2024 Jul 19;18:1368018. doi: 10.3389/fncel.2024.1368018 (PMC11294206; doi:10.3389/fncel.2024.1368018)
Supplement: Supplementary file 1 [file Data_Sheet_1.docx]

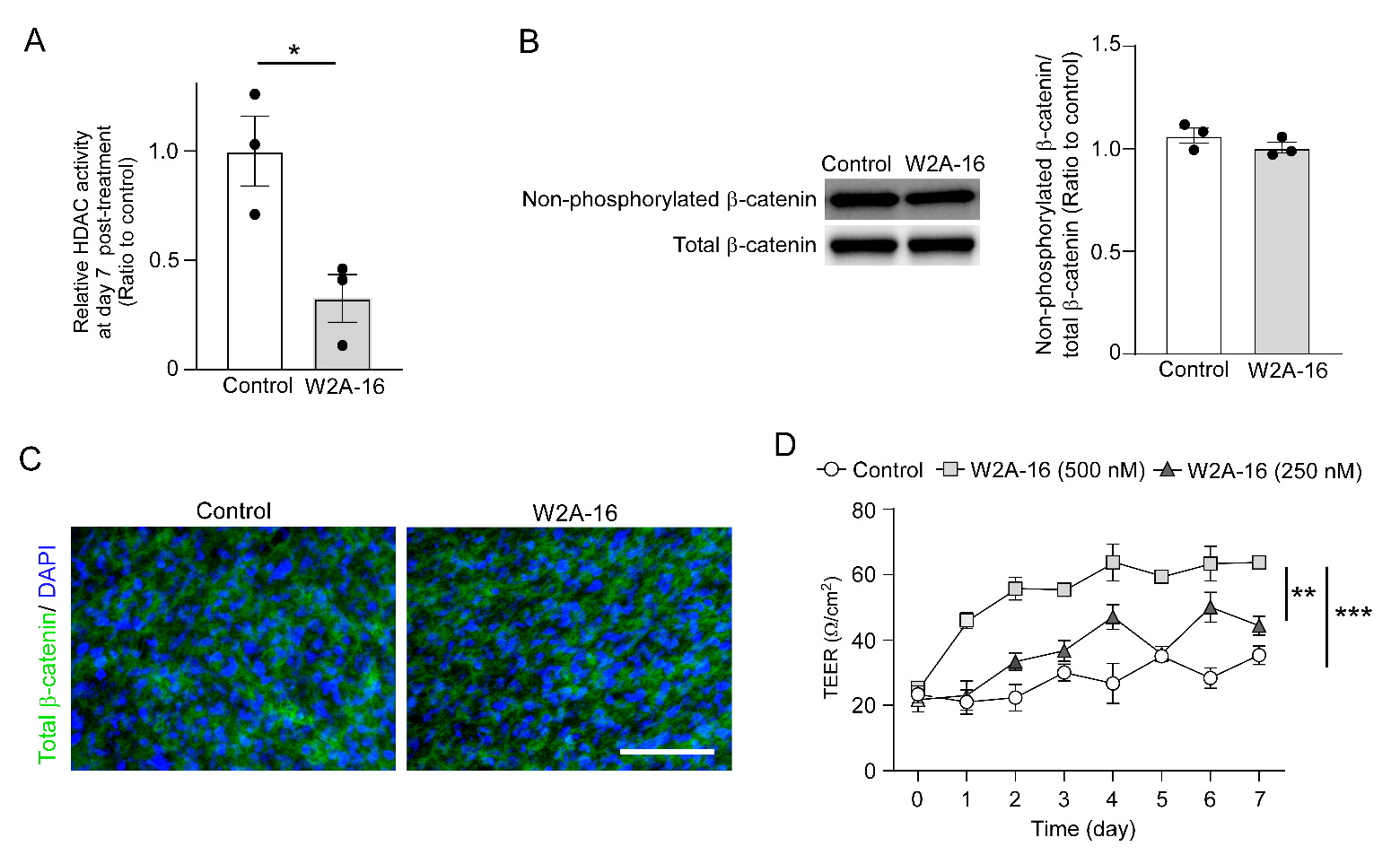


**SUPPLEMENTARY FIGURE 1. Effect of W2A-16 administration on Wnt/β-catenin signaling.**

1. HDAC activity of hCMEC/D3 cells at 7 days post-treatment with W2A-16 (500 nM) or control was assessed by

fluorometric HDAC assay. **(B)** Wnt/β-catenin signaling activity was assessed by measuring activate β-catenin levels through Western blotting after the administration with W2A-16 (500 nM) or vehicle control for 7 days in hCMEC/D3 cells. Data are plotted as mean ± S.E.M. (n= 3/ group). **(C)** Representative images of immunostaining for β-catenin and DAPI in the hCMEC/D3 cells treated with or without W2A-16 for 7 days are shown. Scale bar: 100 μm. **(D)** hCMEC/D3 cells were cultured on the trans-well chambers in the presence of control or W2A-16 (500 nM and 250 nM). TEER was monitored daily for 7 days after the initiation of administration (n= 4/group). Differences between groups were assessed using two-way ANOVA. As vehicle control, 0.025% DMSO was used. Data are plotted as mean ± S.E.M. The two groups were compared by two-tailed Student's t test unless otherwise specified. * p < 0.05, ** p < 0.01, *** p < 0.001.


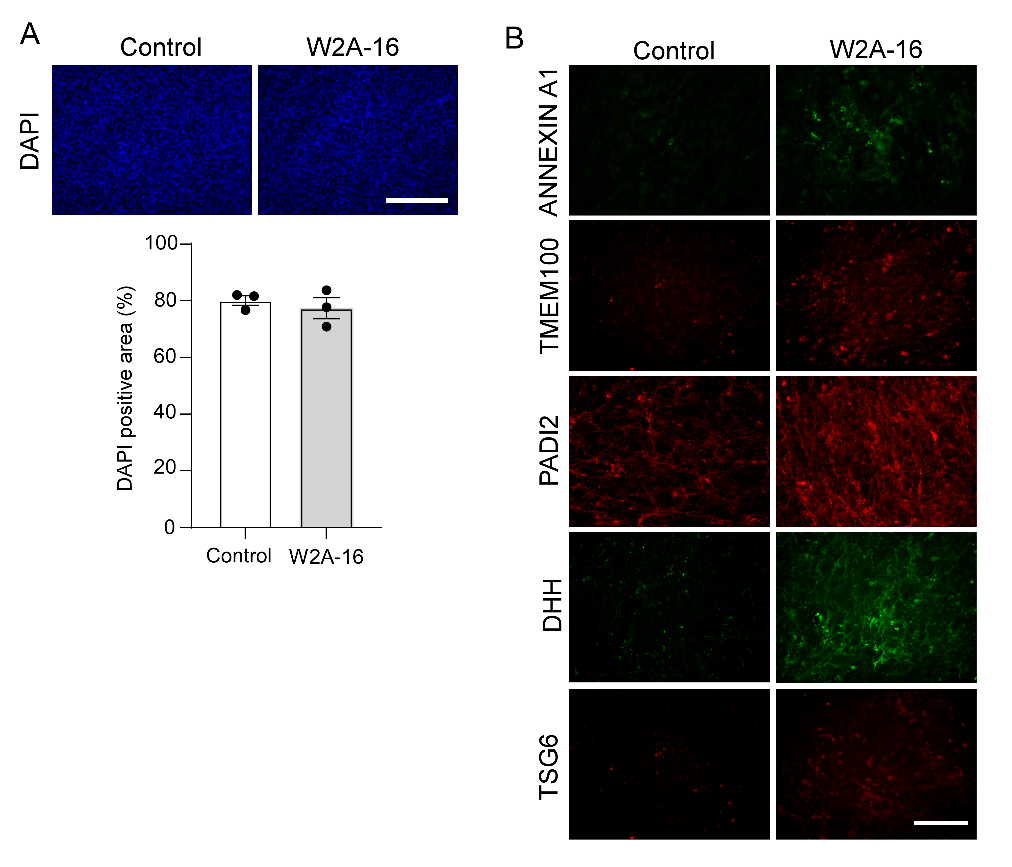


**SUPPLEMENTARY FIGURE 2.**

1. hCMEC/D3 cells were counterstained for DAPI after the administration with W2A-16 (500 nM) or vehicle control for

7 days. Scale bars: 100 μm. **(B)** Representative images of immunostaining for Annexin A1, TMEM100, PADI2, DHH, and TSG6 in the hCMEC/D3 cells treated with or without W2A-16 are shown. Scale bars: 100 μm. As vehicle control, 0.025% DMSO was used. Data are plotted as mean ± S.E.M. The two groups were compared by two-tailed Student's.

**
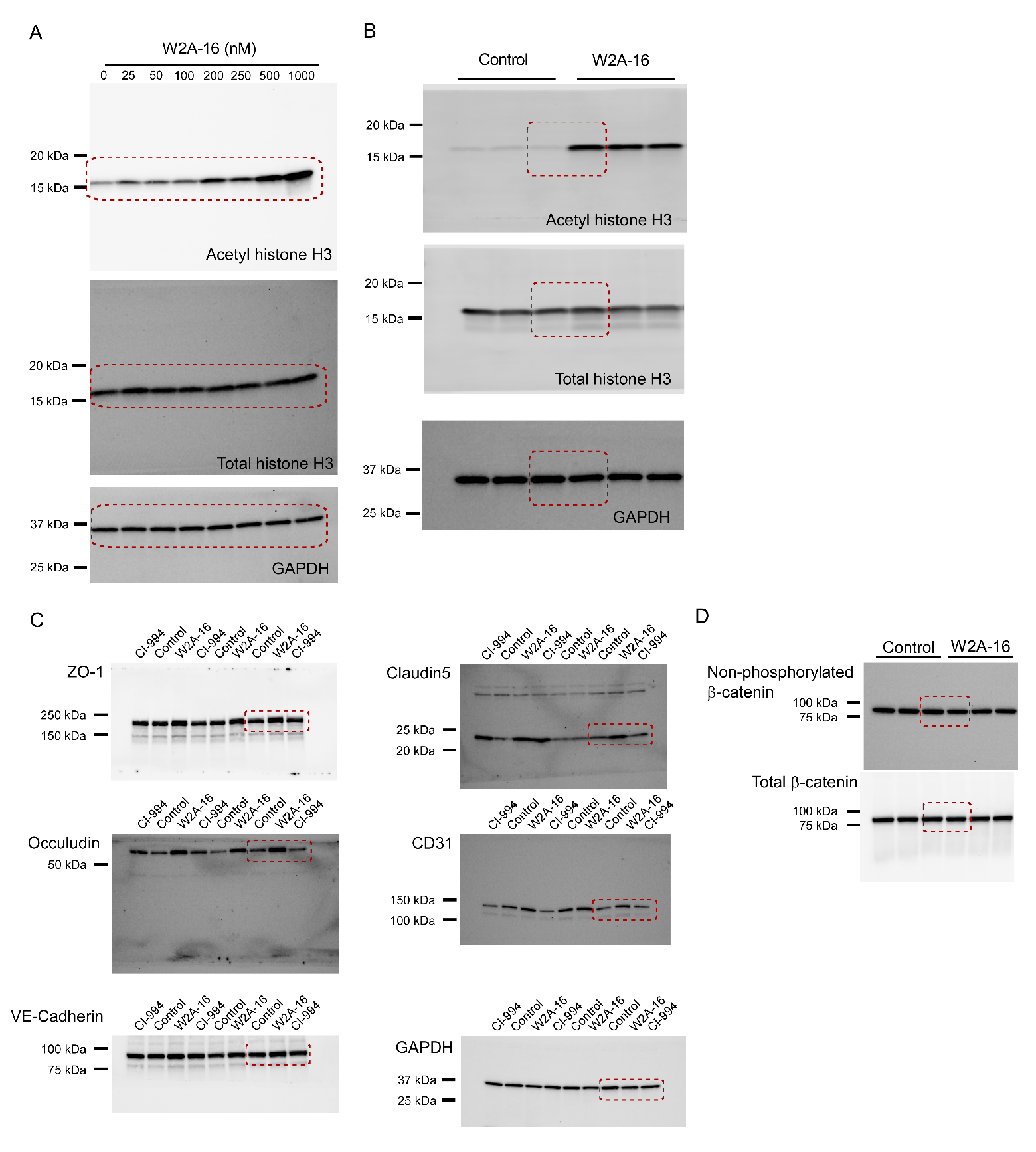
**

**SUPPLEMENTARY FIGURE 3.**

1. Uncropped western blot images of Figure 1A. **(B)** Uncropped western blot images of Figure 1B. **(C)** Uncropped

western blot image of Figure 3A. **(D)** Uncropped western blot images of Supplementary Figure 1B.
